# Isolation of Humic Substances Using Waste Wood Ash Extracts: Multiparametric Optimization via Box–Behnken Design and Chemical Characterization of Products

**DOI:** 10.3390/molecules30153067

**Published:** 2025-07-22

**Authors:** Dominik Nieweś

**Affiliations:** Department of Engineering and Technology of Chemical Processes, Faculty of Chemistry, Wroclaw University of Science and Technology, Wybrzeże Wyspiańskiego 27, 50-370 Wrocław, Poland; dominik.niewes@pwr.edu.pl

**Keywords:** humic substances, humic acids, fulvic acids, wood ash, ultrasound-assisted extraction, Box–Behnken design, FTIR, CP/MAS ^13^C NMR

## Abstract

This study evaluated birch and oak ash extracts as alternative extractants for isolating humic substances (HSs) from peat and lignite. The effects of ultrasound intensity, extraction time, and temperature were optimized using a Box–Behnken design and validated statistically. The highest HSs yields were obtained from peat with oak ash extract (pH 13.18), compared to birch ash extract (pH 12.09). Optimal process parameters varied by variant, falling within 309–391 mW∙cm^−2^, 116–142 min, and 67–79 °C. HSs extracted under optimal conditions were fractionated into humic acids (HAs) and fulvic acids (FAs), and then analyzed by elemental analysis, Fourier Transform Infrared Spectroscopy (FTIR), and Cross-Polarization Magic Angle Spinning Carbon-13 Nuclear Magnetic Resonance Spectroscopy (CP/MAS ^13^C NMR). The main differences in HSs quality were influenced by raw material and fraction type. However, the use of birch ash extract consistently resulted in a higher proportion of carboxylic structures across all fractions. Overall, wood ash extract, especially from oak, offers a sustainable and effective alternative to conventional extractants, particularly for HSs isolation from lignite. Notably, HSs yield from lignite with oak ash extract (29.13%) was only slightly lower than that achieved with 0.5 M NaOH (31.02%), highlighting its practical potential in environmentally friendly extraction technologies.

## 1. Introduction

Achieving high-quality agricultural yields depends on many factors. One of them is the content of soil organic matter (SOM) and its quality, which can deteriorate with the intensification of agricultural production and excessive use of mineral fertilizers. One of the strategies used to prevent this is replenishing the content of high-quality SOM through organic fertilization, which improves soil structure and contributes to maintaining high crop yields in the long term [1,2]. Maintaining the proper quality and quantity of organic matter in the soil not only positively impacts agricultural yield but also contributes to carbon sequestration and the immobilization of pollutants such as heavy metals and pesticides [3,4,5].

All the aforementioned aspects related to the role of organic matter in the soil lead to a continuous increase in interest in organic fertilizers, which is reflected in the growth of the sector’s value. In 2024, it was worth nearly USD 9 billion, with the potential to grow to over USD 16.5 billion by the end of 2034 [6]. Among organic fertilizers, those containing humic substances (HSs) are of particular interest. This is due to the fact that HSs are the main component of SOM, typically accounting for 60 to 80% of the total soil organic matter [7]. It is estimated that by 2034, the value of the humic substance market will double, with a compound annual growth rate (CAGR) of 7.2% [8].

**Figure 1:**
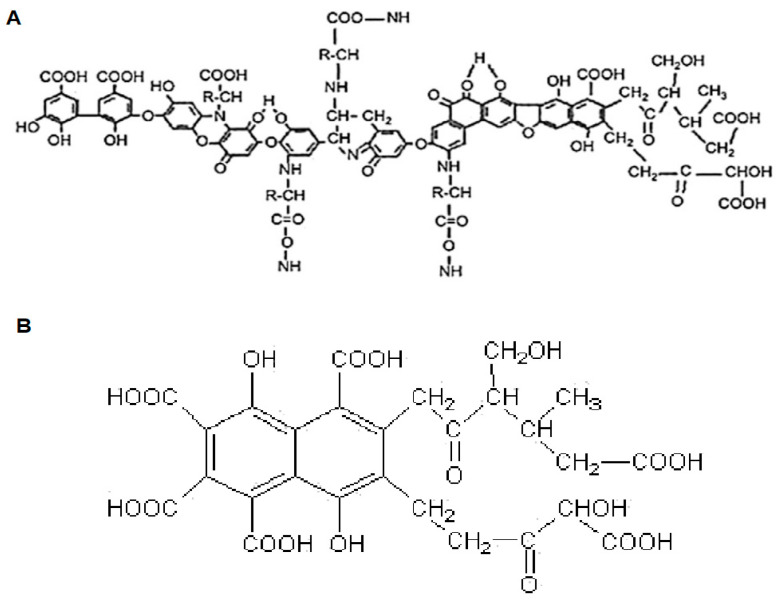
The structural formulas of HAs (**A**) and FAs (**B**) [14,15].

HSs are most often defined as a group of substances with significant variation in both molecular weight and molecular structure, which results from their origin as well as the conditions under which they are formed [9,10]. According to the currently prevailing theory of their structure, HSs are supramolecular structures formed by interactions between small organic molecules containing structures characteristic of lipids, proteins, carbohydrates, or tannins. These structures are stabilized by hydrogen bonds, hydrophobic interactions, or bridging involving cations [11,12]. The most well-known division of humic substances takes into account differences in the solubility of individual fractions depending on pH. Based on this, HSs are divided into fulvic acids (FAs), which are soluble across the entire pH range, humic acids (HAs), which precipitate as a gel from the solution in which they are present at pH < 2, and humin, which is the insoluble fraction [13]. Models of HAs and FAs, illustrating the main differences between these fractions, are shown in Figure 1.

The difference in the solubility of HAs and FAs also forms the basis for the technology of their isolation. The main element of this process is alkaline extraction [16]. The classic raw materials used in this process include peat, lignite, and leonardite [17]. Alternative raw materials include a renewable feedstock, typically waste biomass. It undergoes processes associated with the production of so-called artificial HAs, which include composting and thermal processes [18,19,20]. In the classical approach, the isolation of HSs from raw materials during alkaline extraction is enhanced through mechanical mixing. The mass exchange process can also be enhanced through the application of ultrasound, microwaves, or electric discharges, which enables the production of a larger amount of product from the same mass of raw material, often in less time [21,22,23]. The intended application of isolated HSs is often a determining factor in the choice of extractants and acid solutions used during their extraction and potential fractionation stages. A typical extracting agent is NaOH [24]. However, when the obtained products are intended for fertilization purposes, extracting solutions that supply nutrient elements, such as KOH or NH_3_·H_2_O, may be used [25,26]. This enables the production of a specialized formulation for agricultural purposes, without the need for additional operations after obtaining the HSs that involve, among other procedures, the mixing of isolated humic substances with mineral nutrients to produce organo-mineral fertilizers [27].

The modifications made to the HSs extraction processes so far have focused on increasing process efficiency, as well as on searching for renewable feedstocks that serve as alternatives to the sources previously used [22,28,29]. However, little attention has been given to the use of non-standard extractants, particularly those that could provide a green alternative to the currently employed alkaline solutions. An example of such extractants could be ionic liquids or extracts from wood ash (WAE). Phong et al. (2024) [30] demonstrated the potential application of ionic liquids in isolating fulvic-like substances (FLSs) from wood sawdust. Qualitative analyses revealed that the obtained products were characterized by the presence of, among others, lipid, aromatic, and phenolic structures, which are also characteristic of fulvic acids. The authors also noted the high biological functionality of FLSs, evidenced by their strong radical scavenging activity, thereby indicating the potential use of FLSs not only in agriculture but also in industries such as cosmetics. Furthermore, studies on the potential reuse of ionic liquids were conducted, making the process of isolating FLSs more sustainable [30]. In their review, Sarmah et al. highlight the potential of using aqueous extracts of biomass ash as a green alternative in organic reactions. This approach could eliminate the need for volatile organic compounds [31]. Moreover, the use of wood ash brings a number of benefits, both economic and environmental. Wood ashes may be a source of macro- and micronutrients. Therefore, their use in agriculture and in technologies for producing fertilizer-like products supports nutrient recycling by reintroducing these elements back into the cycle. This helps to close the nutrient loop and also reduces the need for synthetic fertilizers [32,33]. The generation of ash through the combustion of wood waste, in addition to the benefits of agriculturally utilizing the residues from the process, is also associated with energy production. This contributes to increasing the share of energy generation carried out in a sustainable manner [34].

The present study investigates the potential application of aqueous extracts derived from wood ash obtained from birch (*Betula* L.) and oak (*Quercus* L.) in the isolation of HSs from lignite and peat. The quantitative analysis focused on elucidating the relationship between HSs yield and three selected process parameters (ultrasound intensity, time, and temperature), as well as identifying their optimal values to maximize extraction efficiency. Subsequently, HSs isolated under optimized conditions were fractionated into HA FAs. Each fraction was characterized using spectroscopic methods (FTIR, CP/MAS ^13^C NMR) and elemental composition. Analyses were also conducted on the ashes and the extracts obtained from them, including the determination of their pH as well as content of macro- and micronutrient elements.

## 2. Results and Discussion

### 2.1. Characterization of Wood Ash Samples and Their Extracts

The analysis of the ashes and the water extracts obtained from them aimed to determine their suitability, primarily in the context of fertilization. Therefore, it focused on assessing the content of macro- and micronutrients (Table 1), whose presence may have a positive impact on the agricultural use of isolated humic substances, as well as affect the pH of the extracts, which may be relevant to the efficiency of humic substance extraction. The pH values for the birch ash extract and oak ash extract were 12.09 and 13.18, respectively.

The obtained results indicate noticeable differences in the content of macro- and micronutrients in the tested ashes. The ash derived from oak wood was characterized by a higher potassium content, whereas the birch ash exhibited a slightly higher calcium content. Regarding micronutrients, the iron content was dominant compared to the levels of other individual trace elements in both tested ashes, although a higher iron concentration was observed in oak ash. Additionally, it also contained greater amounts of copper and boron, while the birch wood ash showed higher levels of zinc and manganese. However, it should be noted that in the case of micronutrients, they were found in the analyzed ashes mainly in water-insoluble forms, as indicated by their low concentrations in the water extracts. Therefore, their potential effectiveness in the context of fertilization may be limited. Similarly, significant differences in calcium content between the ashes and their water extracts were observed. These large differences are likely due to calcium being present predominantly as calcium carbonate in the ashes, which is practically insoluble in water.

### 2.2. Statistical Analysis

Table 2 presents the results concerning the efficiency of HSs isolation using wood ash extracts for the individual experimental points, which were determined according to the Box–Behnken design, thus forming the experimental matrix. Alongside the coded values of the independent variables, the corresponding decoded values are provided in parentheses. In this study, four responses were obtained. Detailed explanations of the symbols used are provided in Section 3.3.

#### 2.2.1. Effect Estimates of the Tested Variables and Response Surface Plots

The analysis of the responses for individual experimental points in the matrix indicates significant differences in the efficiency of HSs extraction, both due to the type of raw material used and the type of extractant applied. In most cases, higher extraction yields of humic acids were obtained from peat, using both birch and oak ash. When comparing the two types of alkaline extractants used for each raw material (responses Y_1_ vs. Y_2_ and Y_3_ vs. Y_4_), significantly higher extraction yields of HSs were obtained when using the extract derived from oak ash. In some experimental points, the differences were particularly pronounced—for example, in points 4 and 8.

The large differences in extraction yields of HSs, ranging from 7.08% to 36.39%, result not only from variations in the raw material or solvent used but also from differences in the extraction conditions due to the studied parameters (independent variables). Their impact on the obtained responses was determined by the effect values, which are presented in Table 3, Table 4, Table 5 and Table 6. A parameter was considered significant if the *p*-value for the effect was lower than 0.05 [35]. For all evaluated responses, the linear effects of the tested parameters (x_A_, x_B_, x_C_), as well as the quadratic effects related to the intensity of the generated ultrasound (x_A_^2^) and the extraction time (x_B_^2^), had a significant influence on the efficiency of HSs extraction. The quadratic effect of temperature (x_C_^2^) was found to be insignificant (*p* = 0.091) only for the extraction of HSs from lignite using birch ash extract as the solvent (response Y_3_). Among the tested interactions between extraction parameters, only the isolation of HSs from peat using birch ash extract (response Y_1_) showed two significant effects out of three. These were the interactions between ultrasound intensity and extraction time (x_A_∙x_B_), as well as ultrasound intensity and process temperature (x_A_∙x_C_).

**Figure 2:**
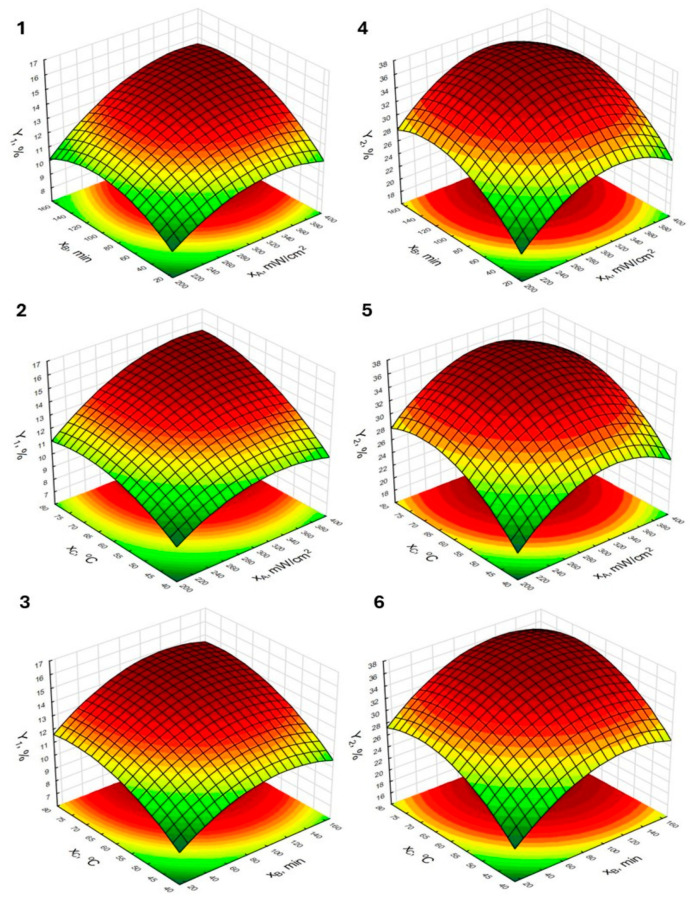
Response surfaces and contour plots for the HSs isolation from peat using birch (**1**–**3**) and oak (**4**–**6**) ash extract.

**Figure 3:**
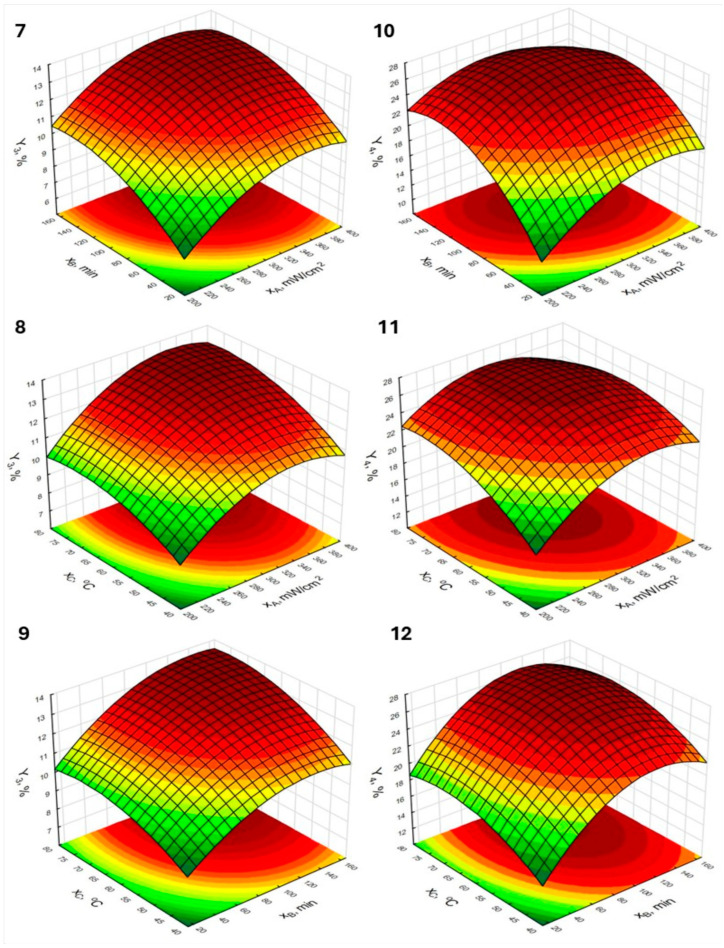
Response surfaces and contour plots for the HSs isolation from lignite using birch (**7**–**9**) and oak (**10**–**12**) ash extract.

The influence of the studied independent variables on the evaluated responses was visualized using response surface plots along with contour plots, shown in Figure 2 and Figure 3, and the responses were grouped according to the type of raw material from which the HSs were isolated. Most of the plots exhibit a parabolic shape, which indicates that the optimal process conditions were obtained within the investigated experimental space [36]. This is particularly evident in the plots labeled 4–6 and 10–12, which correspond to extractions, where oak ash extract was used as the extracting agent. For these processes (responses Y_2_ and Y_4_), the quadratic effects were markedly higher compared to the processes utilizing birch ash extract (responses Y_1_ and Y_3_). When comparing the plots illustrating the efficiency of HSs extraction from peat using birch ash extract as the extractant, it can be observed that the plots labeled 1 and 2 exhibit slightly greater curvature. This may result from the significance of the interaction effect between ultrasound intensity and extraction time, as shown in plot 1, as well as between ultrasound intensity and temperature, as illustrated in plot 2. The response surface representing Y_1_ as a function of extraction time and temperature (plot 3) is flatter at higher yield levels, which is consistent with the lack of significance of the interaction effect between the variables presented as independent factors in this plot. There is a noticeable similarity in the shapes of plots 1–3 (response Y_1_) and plots 7–9 (response Y_3_) as well as between plots 4–6 and 10–12, which illustrate responses Y_2_ and Y_4_, respectively. Based on this observation, it can be concluded that the response patterns and the influence of the studied independent parameters are also determined by the type of extractant used, whereas the type of raw material from which the HSs were isolated may be associated with significant differences in extraction efficiency under identical extraction conditions. In this study, peat was a richer source of humic substances (HSs) compared to lignite, which is supported by some of the data presented in the literature [37,38]. Wu et al. [37] indicate that, depending on the type of peat, the yield of humic acid extraction can exceed 50%. At the same time, the results for various types of lignite presented by Zara et al. [38] show that the extraction yield of humic acids from the samples tested in their study did not exceed 25%. Differences in the content of humic substances may result from varying conditions under which the humification process occurs (i.e., temperature, moisture) as well as differences in microbial activity, which affect the degree of the transformation of the original organic matter [39,40]. Interesting conclusions regarding the analyzed effects may also be drawn from the comparison of the response surfaces related to response Y_4_, specifically those shown in plots 10, 11, and 12. In this case, a noticeable flattening can be observed in plots 10 and 11, whereas plot 12 exhibits greater curvature, which presents a certain analogy to the plots corresponding to response Y_1_ and is related to interaction effects. Although the interaction effects for response Y_4_ were not statistically significant, the *p*-values for the effects of x_A_∙x_B_ and x_A_∙x_C_ were close to the significance threshold (0.056 and 0.087, respectively).

#### 2.2.2. Modeling and Optimization

The statistically significant effects formed the basis for generating polynomial equations that described the relationships between the responses and the tested independent variables. Based on these equations, it was possible to identify the optimal values of ultrasound intensity, process duration, and extraction temperature to maximize the yield of HSs, depending on the raw material and the extraction system used. These models are presented as Equations (1)–(4). They refer to the coded forms of the independent variables, and the coefficients represent half the values of the effects for the respective parameters.

Y_1_ = 11.25 + 1.58 · x_A_ + 1.24 · x_B_ + 1.76 · x_C_ + 0.70 · x_A_^2^ + 0.79 · x_B_^2^ + 0.61 · x_C_^2^ + 0.62 · x_A_ · x_B_ + 0.60 · x_A_ · x_C_ (1)

Y_2_ = 26.53 + 2.29 · x_A_ + 3.74 · x_B_ + 3.83 · x_C_ + 2.50 · x_A_^2^ + 2.08 · x_B_^2^ + 2.08 · x_C_^2^ (2)

Y_3_ = 10.52 + 1.37 · x_A_ + 1.47 · x_B_ + 0.99 · x_C_ + 0.73 · x_A_^2^ + 0.57 · x_B_^2^ (3)

Y_4_ = 19.90 + 1.58 · x_A_ + 3.24 · x_B_ + 1.78 · x_C_ + 1.62 · x_A_^2^ + 2.18 · x_B_^2^ + 1.05 · x_C_^2^ (4)

**Figure 4:**
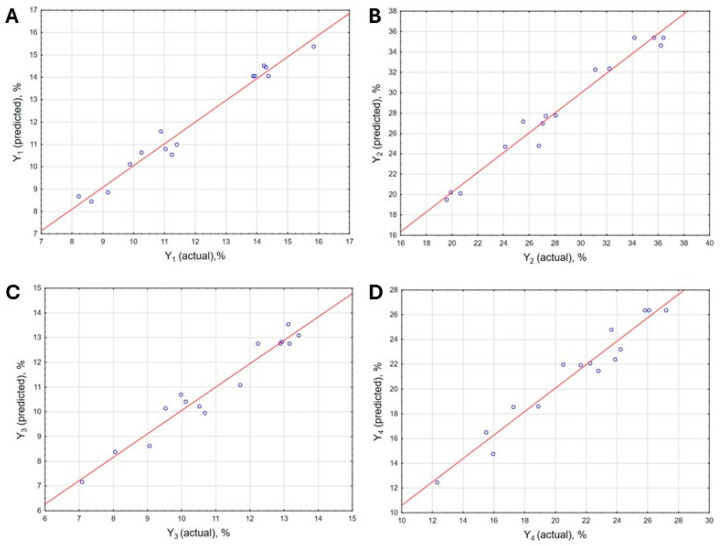
Predicted vs. actual results for the efficiency of HSs isolation from peat (**A**,**B**) and lignite (**C**,**D**).

The analysis of variance (ANOVA) for the individual equations is presented in Table 7. The fit of the models to the experimental data was evaluated based on the lack-of-fit test, as well as analysis using Fisher’s test (F-test), taking into account the Fisher–Snedecor distribution at a significance level of 0.05. Additionally, the determination coefficients (R^2^) were assessed, and a graphical comparison between the predicted and actual results was performed (Figure 4).

For all presented models, the *p*-values for the lack-of-fit test were greater than 0.05. This indicates that, in each of the considered cases, the proposed polynomial equations accurately predict the actual results. Additionally, for each of the models, the calculated F-values (F_cal_.) were higher than the tabulated value for the considered experimental design, which in this case was 4.77 [41]. The goodness of fit of the models to the experimental data is also supported by the determined coefficients of determination (R^2^). For the models describing responses Y_1_ and Y_2_, the R^2^ values were identical and amounted to 97.12%. In the case of Equations (3) and (4), which refer to the extraction of HSs from lignite, the R^2^ values were slightly lower, amounting to 89.86% for Equation (3) and 89.43% for Equation (4). According to Manzato et al., a coefficient of determination exceeding 80% indicates a good model fit [42]. In the present study, all of the generated models exceeded this threshold, suggesting that they appropriately reflect the actual results. The described differences between the models are also visible in the plots illustrating the predicted vs. observed results. In the case of the plots for the models related to HSs extraction from peat (Figure 4A,B), the arrangement of the experimental points is generally closer to the model line compared to Figure 4C,D, which correspond to the processes of HSs isolation from lignite. These discrepancies, driven by differences in the raw materials used rather than the type of extractant, indicate that the efficiency of HSs extraction from lignite is more difficult to predict using design of experiments and response surface methodology.

Based on the generated models, a process simulation was conducted, and the optimal conditions for ultrasound-assisted extraction were identified depending on the type of raw material and extractant used, as presented in Table 8. The predicted results were subsequently validated under experimental conditions. A comparison of the optimal predicted yields indicates that the differences in response for the individual variants in this case were primarily determined by the type of extractant used. This may be attributed to the higher alkalinity of oak ash, as confirmed by the measured pH values of the extracts (Section 2.1) [43]. Considering the optimal predicted results in relation to the type of raw material, higher yields were obtained when HSs were extracted from peat. The use of oak ash extract further increased the yield difference depending on the raw material used.

All of the determined optimal values of the independent variables fell within the tested ranges, which may indicate that the experimental space was well-designed and that the extremum of the dependent variable was achieved within this range for all considered cases. Comparing the determined values for the individual processes, it can be observed that the maximum yield of HSs isolation was achieved more quickly, at lower temperatures, and with lower ultrasound intensity when oak ash extract was used as the extractant. This trend was consistent regardless of the type of raw material from which the HSs were extracted. The verification of the theoretical maximum efficiencies under laboratory conditions indicated that, in each case, the actual results were slightly higher than the predicted values; however, the observed differences were not significant.

The extraction yields of HSs presented in Table 8 were compared with the results obtained using NaOH solutions, which are considered a classical extractant (Table 9). The extraction of HSs from peat and lignite using 0.1 M and 0.5 M NaOH solutions was conducted under conditions optimized for the extraction of HSs with oak ash extract. These conditions were selected because the oak ash extract provided better yields than the second tested extractant.

The extraction efficiency of HSs using NaOH was higher in all cases compared to the efficiency achieved with wood ash extracts. The extraction efficiency was higher for peat, which was also observed when comparing the tested raw materials in the context of extraction using wood ash extracts. The efficiency of extraction also increased with the concentration of the extractant. Yang et al. (2024) observed a significant increase in the efficiency of humic acid extraction from lignite with increasing NaOH concentration, specifically within the range of 0.15 to 0.30 mol/dm^3^, during their investigation of the influence of extraction process parameters [44]. This can be attributed to the higher pH of these solutions, which promoted the increased solubility of the humic acid fraction [45]. However, when lignite was used as the raw material, the differences were relatively small. Considering that wood ash extracts can also serve as a source of potassium, this may indicate their potential for use in the extraction of HSs from lignite for fertilizer applications.

### 2.3. Qualitative Assessment

These samples were labeled in accordance with the designations used for the respective variants in the quantitative analysis.

#### 2.3.1. Elemental Composition

The elemental composition of HAs and FAs are presented in Table 10. The results indicate that both humic and fulvic acids isolated from peat are characterized by lower carbon content and, simultaneously, higher heteroatom content. This may lead to the conclusion that samples extracted from peat may be richer in functional groups containing nitrogen and oxygen, which is also related to the degree of humification [46]. Through the comparison of the individual fractions obtained from the same raw material and use of the same extractant, it can be noted that the fulvic acid (FA) fraction is characterized by higher content of heteroatoms (nitrogen and sulfur), whereas the humic acids (HAs) exhibit a greater proportion of carbon atoms in their molecular structure. This may indicate a higher hydrophobicity of the humic acid fraction, as well as a greater contribution of aromatic structures in their molecular composition [47,48]. In analyzing the results with regard to the type of extractant used, the differences in elemental composition are generally insignificant. This suggests that the differences in composition are primarily determined by the type of raw material from which the samples were obtained and the type of fraction evaluated.

#### 2.3.2. FTIR

**Figure 5:**
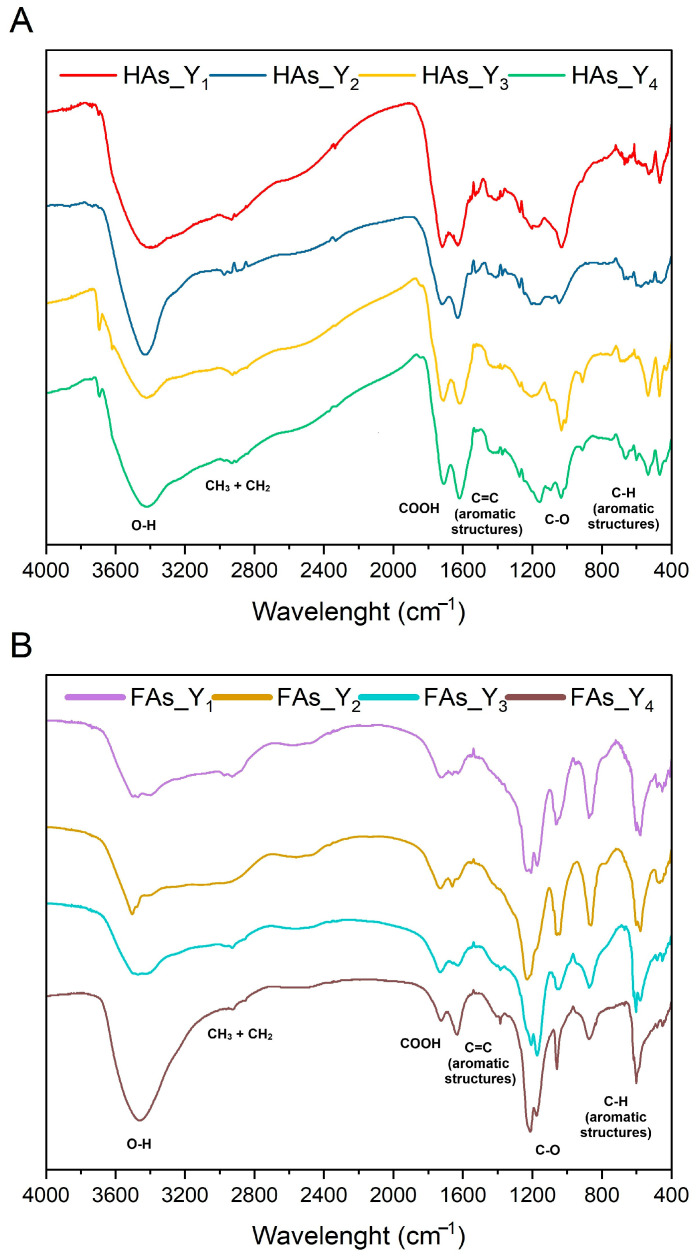
FTIR spectra of humic (**A**) and fulvic (**B**) acids analyzed in this study.

The analysis of FTIR spectra of the samples (Figure 5) enabled the identification of molecular structures characteristic of humic substances, including key functional groups that determine their properties. The broad band centered at approximately 3400 cm^−1^ corresponds to O–H stretching vibrations in phenols, alcohols, and water [49]. The weak peaks observed at 2920 and 2850 cm^−1^ indicate C–H stretching vibrations of methyl and methylene groups in aliphatic chains [50]. The peak at 1720 cm^−1^ and the band centered around 1600 cm^−1^ are attributed to C=O stretching in carboxylic groups and C=C vibrations in aromatic structures, respectively [51]. Signals between 1200 and 1000 cm^−1^ are related to C–O stretching in esters, polysaccharides, and alcohols [52]. Bands in the 900–700 cm^−1^ region may be assigned to out-of-plane C–H bending in aromatic structures [53].

#### 2.3.3. CP/MAS ^13^C NMR

**Figure 6:**
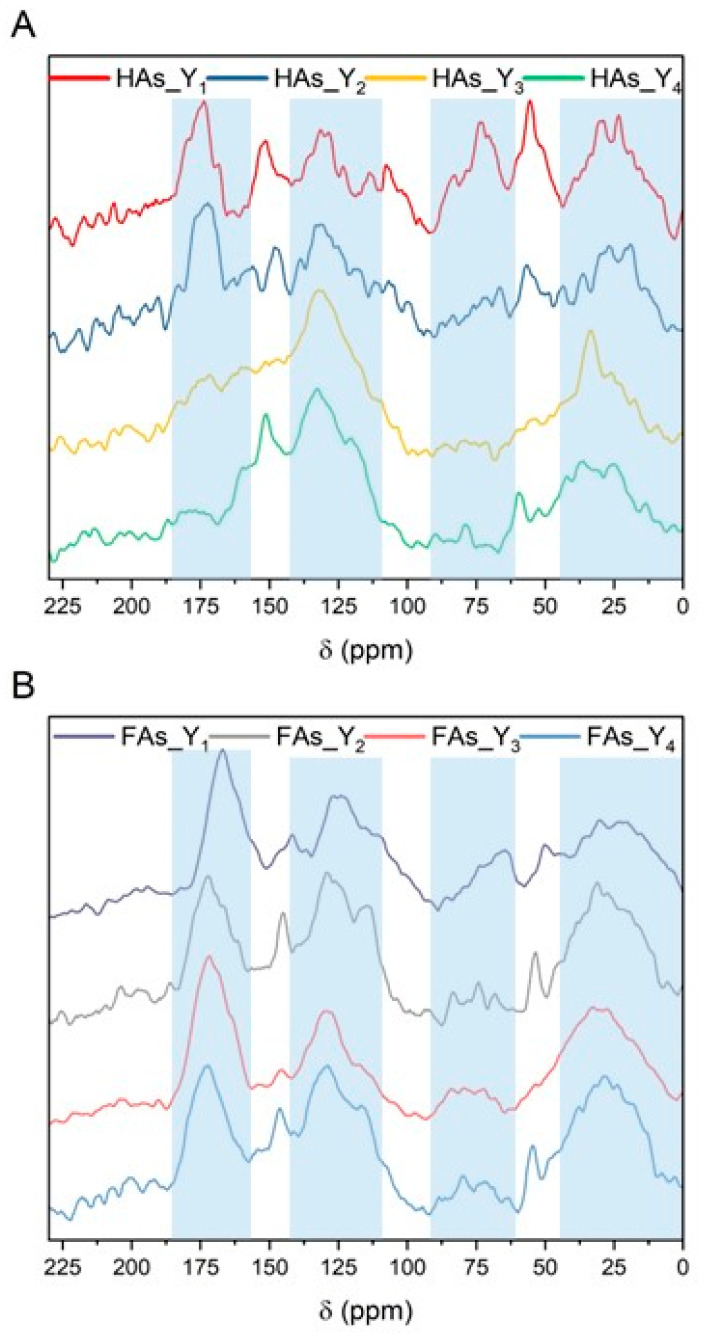
CP/MAS ^13^C NMR spectra of humic (**A**) and fulvic (**B**) acids isolated.

The acquisition and analysis of the CP/MAS ^13^C NMR spectra of the studied samples (Figure 6) aimed to determine the differences in the contribution of carbon atoms within specific structures in the analyzed humic and fulvic acids. By analyzing the spectra, one can observe maxima in the resonance regions, which are characteristic of structural features typical for humic and fulvic acids. The broad peak with a maximum around 30 ppm is associated with the presence of methyl and methylene groups in aliphatic structures [54]. A small signal around 50 ppm, characteristic mainly of fulvic acid samples, may be attributed to methoxy groups; however, in this region, a peak indicating a CH–N bond—potentially present in proteins—may also appear [55]. The signal at 130 ppm originates from aromatic, unsaturated carbon, while the signal at 150 ppm corresponds to carbon atoms bonded to phenols, as well as aromatic rings substituted with oxygen or nitrogen [56]. Finally, the peak at 173 ppm, may be assigned to carboxyl carbon, as well as the carbonyl carbon of amides and polypeptides [57].

Through the analysis of the results related to the percentage share of carbon atoms in specific molecular structures of the evaluated samples (Table 11), it can be concluded that these differences were primarily due to the type of raw material from which the samples were isolated (peat or lignite), as well as the type of evaluated fraction (humic acids or fulvic acids). However, certain differences were also observed depending on the type of extractant used.

In general, the dominant forms of carbon in all samples were aliphatic and aromatic carbon not bonded to heteroatoms, although the samples also exhibited a relatively high proportion of carboxylic carbon. When comparing the samples based on the type of fraction, it can be observed that humic acids typically contained a higher proportion of aromatic carbon. The fulvic acid fractions was richer in oxygen-substituted alkyl structures and showed a higher content of carboxylic carbon. These comparisons suggest that the humic acid fraction is more aromatic than the fulvic acid fraction, which aligns with previous findings [58]. Regarding the type of raw material, samples derived from lignite contained higher proportions of aromatic and non-substituted alkyl carbon (not bonded to heteroatoms), whereas the fractions isolated from peat generally exhibited a higher content of heteroatoms in their molecular structure. Considering the type of extractant used, the application of oak ash extract resulted in a higher content of carbonyl carbon and a lower content of carboxyl carbon in the obtained HAs. In the case of FAs, it was observed that the use of birch ash extract led to a higher content of carboxyl carbon.

## 3. Materials and Methods

### 3.1. Chemicals and Raw Materials

The wood ash samples used in this study were obtained from birch (*Betula* L.) and oak (*Quercus* L.) sawdust, sourced from a local sawmill. Humic substances were isolated from lignite originating from the Szczerców deposit and from peat collected from a peatland located in the Vistula River estuary region. The 6 M HCl solution used for the precipitation of the humic acid fraction was prepared by diluting concentrated hydrochloric acid (38% *w*/*w*). A 0.1 M NaOH solution was prepared using an accurately weighed portion of analytical-grade sodium hydroxide. All chemicals were purchased from Chempur (Piekary Śląskie, Poland). Deionized water with conductivity below 1 µS/cm was used to prepare all solutions, including the extracts obtained from the wood ash samples.

### 3.2. Analysis of Wood Ash Samples and Their Extracts

The analysis of wood ashes and their aqueous extracts included the determination of macro- and micronutrients relevant to fertilization. Elemental concentrations were determined using inductively coupled plasma optical emission spectrometry (ICP-OES 5800, Agilent Technologies, Santa Clara, CA, USA) based on a calibration curve method. Prior to measurement, ash samples were subjected to mineralization. For this purpose, 500 mg of wood ash was placed in tightly sealed PTFE digestion vessels, to which 5 cm^3^ of concentrated nitric acid (69% *w*/*w*, Merck, Suprapur, Darmstadt, Germany) was added. The vessels were then heated in a microwave digestion system (Milestone START D, Sorisole, Italy) at 200 °C with a ramp time of 10 min and held at this temperature for 20 min, followed by cooling. After pressure release, the digested solutions were transferred into polypropylene bottles, diluted with deionized water to a total weight of 50 g, and subsequently analyzed. In the case of aqueous extract analysis, prior to elemental determination, the extracts were acidified by mixing 9 cm^3^ of the extract with 1 cm^3^ of concentrated nitric acid (V). This step was carried out to ensure matrix matching with other samples and calibration standards.

### 3.3. Isolation and Fractionation Procedure for Humic Substances

The first step of the research involved obtaining extracts from wood ash, which were subsequently used as alkaline solutions for the isolation of humic substances (HSs) from peat and lignite. To this end, birch and oak sawdust was first dried at 105 °C for 24 h and then incinerated in a muffle furnace at 700 °C for 5 h. The resulting ash was transferred into Erlenmeyer flasks and mixed with deionized water at a mass ratio of 1:10. The mixtures were subjected to ultrasonic treatment at an intensity of 400 mW∙cm^−2^ for 120 min at a temperature of 80 °C. Following the treatment, the extract was separated from the solid phase by vacuum filtration using a hard qualitative filter. The resulting clear solution was cooled to room temperature and stored in glass bottles for further use.

The procedure related to the isolation, quantitative assessment, and fractionation of the extract containing HAs and FAs was based on the guidelines of the International Humic Substances Society (IHSS) concerning the extraction of humic substances (HSs) from solid raw materials, as well as ISO standards 19822:2018 and 5073:2021 [59,60,61]. However, in relation to the aforementioned methods, certain modifications were introduced in this study to adapt the HSs isolation process with the aim of maximizing extraction efficiency and enabling quantitative evaluation in the context of parameters whose effects were tested according to the Box–Behnken design. Initially, pre-crushed peat and lignite were dried at 105 °C for 24 h and subsequently ground to particles with a diameter of less than 1 mm. Ten grams of the prepared material were transferred to Erlenmeyer flasks and mixed with 100 cm^3^ of ash extracts. The isolation of humic substances (HSs) was performed using ultrasound-assisted extraction in a thermostated ultrasonic bath EMMI 40 HC (EMAG, Salach, Germany), operating at a frequency of 45 kHz. The ultrasound intensity, extraction time, and temperature were variable and dependent on the specific experimental point within the matrix, as described in detail in Section 2.1 and Section 3.3. Subsequently, the solid phase was separated by centrifugation (4000 rpm for 10 min), and the solid particles remaining in the liquid were removed by vacuum filtration using the hard qualitative filter. The resulting HSs extract was quantitatively transferred to porcelain evaporating dishes and dried at 105 °C to a constant weight. The next step involved incinerating the obtained HSs at 650 °C for 5 h, which allowed for the determination of the HSs extraction yield, calculated as the mass ratio of the obtained HSs to the mass of the raw material used, expressed on a dry and ash-free basis.

The quantitative studies of the extraction of humic substances (HSs) aimed to identify the optimal conditions for the process to achieve maximum extraction efficiency. Subsequently, the HSs isolated under these conditions were fractionated into humic acids (HAs) and fulvic acids (FAs). For this purpose, the obtained HSs extract was acidified with a 6 M HCl solution to pH 1 and then stored at 5 °C for approximately 16 h, ensuring complete precipitation of the HAs fraction. The precipitated HAs were then separated from the liquid phase, which contained the FAs fraction, by gravity filtration using qualitative filters. The resulting HAs gel was dried at 105 °C for 24 h. The FAs were isolated from the solution following the precipitation HAs. The process involved the adsorption of FAs onto a hydrophobic resin (Supelite DAX-8, Sigma-Aldrich, St. Louis, MO, USA). For this purpose, the FAs-containing solution was passed through a resin-packed column at a constant flow rate of 4.5 cm^3^·min^−1^. After the solution had passed through, the column was rinsed with deionized water to remove unbound substances. The FAs were then eluted by backflushing the column with 0.1 M NaOH, delivered at the same flow rate. The eluate was subsequently protonated by passing it through a column packed with acidic ion-exchange resin. To ensure complete protonation of the FAs, this step was repeated five times. The resulting solution was concentrated to approximately 50 cm^3^ using a rotary evaporator (Buchi, Flawil, Switzerland). Finally, the FAs concentrate was dried at 105 °C to a constant mass.

### 3.4. Quantitative Assessment

The quantitative assessment of the processes of HSs isolation focused on determining the influence of ultrasound intensity (x_A_), extraction time (x_B_), and temperature (x_C_) on the yield of humic substances (HSs) isolated from peat and lignite using aqueous extracts of oak and birch ash. Each of the tested independent variables was coded at three levels, with −1 representing the minimum value; 0, the central value; and 1, the maximum value of the given parameter within the evaluated experimental space. For ultrasonic intensity, the values used were 200, 300, and 400 mW∙cm^−2^; for the extraction time of humic substances, the values were 15, 90, and 165 min; and for temperature, the adopted values were 40, 60, and 80 °C. The four responses were obtained in this study, all corresponding to the yield of humic substance isolation. The differences between them resulted from the type of raw material used (peat or lignite) and the type of extraction solution applied (birch ash extract or oak ash extract). Accordingly, the following were evaluated:

- The yield of HSs isolated from peat using birch ash extract (Y_1_);
- The yield of HSs isolated from peat using oak ash extract (Y_2_);
- The yield of HSs isolated from lignite using birch ash extract (Y_3_);
- The yield of HSs isolated from lignite using oak ash extract (Y_4_).

The experimental matrix was designed based on the Box–Behnken design (BBD). This required conducting 15 experiments for each evaluated process, including a central point, which was tested in triplicate. The experimental points in the matrix were carried out in a random order to reduce noise and minimize the influence of unaccounted variables on the generated results.

### 3.5. Qualitative Analysis of Humic Substances

Samples of humic substances, which were isolated under quantitatively optimal process conditions for each variant, were separated into humic acids (HAs) and fulvic acids (FAs) fractions and subsequently subjected to qualitative analysis. In this part of the study, four humic acids samples and four fulvic acids samples were examined. Accordingly, the samples labeled as HAs_Y_1_ and FAs_Y_1_ represent the humic acids and fulvic acids fractions, respectively, obtained from peat using birch ash extract. HAs_Y_2_ and FAs_Y_2_ correspond to humic and fulvic acids extracted from peat using oak ash extract. The designations HAs_Y_3_ and FAs_Y_3_ refer to humic and fulvic acids fractions isolated from lignite using birch ash extract. Finally, samples HAs_Y_4_ and FAs_Y_4_ represent the humic and fulvic acids fractions extracted from lignite using oak ash extract. This study included elemental composition analysis (CHNSO) and various spectroscopic techniques, including Fourier Transform Infrared Spectroscopy (FTIR) and Cross-Polarization Magic Angle Spinning Carbon-13 Nuclear Magnetic Resonance (CP/MAS ^13^C NMR).

The percentage contents of carbon (C), hydrogen (H), nitrogen (N), and sulfur (S) were determined using a Vario EL Cube elemental analyzer (Elementar, Langenselbold, Germany). Acetanilide and sulphanilamide were used as calibration standards. For each sample, three replicates were analyzed. The results were calculated on a dry, ash-free basis and expressed as mean values with standard deviations. The oxygen (O) content was determined by the difference.

FTIR spectra were recorded at room temperature using a Bruker Vertex 70 spectrometer (Billerica, MA, USA). To prepare the samples, humic and fulvic acids were mixed with KBr at a mass ratio of 1:100 and pressed into pellets. Spectra were collected in the wavenumber range of 4000 to 400 cm^−1^ with a resolution of 4 cm^−1^.

CP/MAS ^13^C NMR measurements of solid samples were performed using a Bruker Avance III 300 spectrometer equipped with a 4 mm wide-bore MAS probe. The instrument operated at a resonance frequency of 75.45 MHz, with a rotor spinning speed of 5 kHz. Each spectrum was recorded with an acquisition time of 20 ms and a recycle delay of 3 s. Data were collected over a chemical shift range of 0 to 230 ppm, and tetramethylsilane (TMS) was used as the reference standard. All measurements were conducted at room temperature. Therefore, in accordance with the information provided by Li et al., the studied resonance region was divided into eight zones (Table 9) corresponding to the contribution of carbon atoms in aliphatic structures (0–45 ppm), chains bonded to nitrogen or oxygen atoms (45–60 ppm), aliphatic structures in which the carbon atom is bonded to one (60–91 ppm) or two (91–110 ppm) oxygen atoms, unsubstituted aromatic structures (110–142 ppm), rings with a carbon atom bonded to oxygen or nitrogen (142–156 ppm), and carboxylic (156–186 ppm) and carbonyl structures (186–230 ppm) [62].

## 4. Conclusions

The conducted research demonstrated that aqueous extracts derived from wood ash, particularly from oak, represent a promising and sustainable alternative to conventional alkaline solutions for the isolation of HSs from a quantitative perspective. Under the identified optimal process conditions, higher extraction yields of HSs were achieved using oak ash extract, while the process also required lower ultrasound intensity, temperature, and extraction time, enhancing its practical applicability.

From a qualitative standpoint, the type of extractant had a limited impact on the molecular structure of the obtained humic fractions. However, it was observed that birch ash extract promoted the formation of fractions with a higher carboxyl carbon content, which may be relevant in the context of sorption properties and interactions with nutrient elements in soil. Spectroscopic and elemental analyses confirmed the presence of structural features typical of HSs, including aliphatic chains, aromatic rings, and carboxylic groups.

In summary, oak ash extract enables higher yields of HSs isolation, whereas birch ash may contribute to more favorable chemical characteristics of the resulting fractions. These findings confirm the potential of using wood ashes to produce alkaline extracts as environmentally friendly solutions that can be considered in HSs extraction technologies.

## Figures and Tables

**Table 1:** Contents of selected macro- and microelements of oak and birch wood ash and their water extracts.

Element	Unit	Birch Wood Ash	Oak Wood Ash	Birch Wood Ash Extract	Oak Wood Ash Extract
K	mg∙g^−1^	122.01	152.10	40.56	54.07
Ca	mg∙g^−1^	289.14	280.70	0.46	0.78
Cu	ppm	209.21	324.43	0.02	0.02
Fe	ppm	1680.74	2412.41	0.02	0.01
Zn	ppm	366.93	207.38	0.02	0.04
B	ppm	630.00	819.60	9.98	9.31
Mn	ppm	189.00	177.90	0.03	0.02

**Table 2:** Experimental matrix used in this study, designed according to the Box–Behnken design for three independent variables.

Run	Independent Variables ^1^			Responses ^2^			
	x_A_	x_B_	x_C_	Y_1_	Y_2_	Y_3_	Y_4_
1	−1 (200)	−1 (15)	0 (60)	9.16	19.90	7.08	12.30
2	1 (400)	−1 (15)	0 (60)	11.03	26.72	10.52	18.89
3	−1 (200)	1 (165)	0 (60)	9.88	27.28	10.12	21.63
4	1 (400)	1 (165)	0 (60)	14.23	31.13	12.93	22.26
5	−1 (200)	0 (90)	−1 (40)	8.21	20.63	8.05	15.49
6	1 (400)	0 (90)	−1 (40)	10.25	24.11	9.98	20.51
7	−1 (200)	0 (90)	1 (80)	11.40	28.03	10.68	23.87
8	1 (400)	0 (90)	1 (80)	15.84	32.21	13.43	24.23
9	0 (300)	−1 (15)	−1 (40)	8.62	19.58	9.06	15.94
10	0 (300)	1 (165)	−1 (40)	11.24	27.03	11.71	22.79
11	0 (300)	−1 (15)	1 (80)	10.89	25.53	9.52	17.25
12	0 (300)	1 (165)	1 (80)	14.29	26.21	13.12	23.63
13	0 (300)	0 (90)	0 (60)	13.93	35.68	12.89	26.07
14	0 (300)	0 (90)	0 (60)	13.88	34.16	12.23	25.80
15	0 (300)	0 (90)	0 (60)	14.37	36.39	13.16	27.19

^1^ Independent variables: x_A_—ultrasound intensity; x_B_—time; x_C_—temperature. ^2^ Responses: Y_1_—the yield of HSs isolation from peat using birch ash extract; Y_2_—the yield of HSs isolation from peat using oak ash extract; Y_3_—the yield of HSs isolation from lignite using birch ash extract; Y_4_—the yield of HSs isolation from lignite using oak ash extract.

**Table 3:** Effect estimates of the independent parameters on the efficiency of HSs isolation from peat using birch ash extract (response Y_1_).

Source	Effect	Standard Error	Confidence Interval		*p*-Value	Remarks
			+95%	−95%		
x_A_	3.17	0.19	2.35	4.00	0.004	Significant
x_B_	2.49	0.19	1.66	3.31	0.006	Significant
x_C_	3.52	0.19	2.70	4.35	0.003	Significant
x_A_^2^	1.41	0.14	0.81	2.01	0.010	Significant
x_B_^2^	1.57	0.14	0.97	2.18	0.008	Significant
x_C_^2^	1.22	0.14	0.62	1.83	0.013	Significant
x_A_∙x_B_	1.24	0.27	0.08	2.40	0.044	Significant
x_A_∙x_C_	1.20	0.27	0.04	2.36	0.047	Significant
x_B_∙x_C_	0.39	0.27	−0.77	1.55	0.285	Non-significant

**Table 4:** Effect estimates of the independent parameters on the efficiency of HSs isolation from peat using oak ash extract (response Y_2_).

Source	Effect	Standard Error	Confidence Interval		*p*-Value	Remarks
			+95%	−95%		
x_A_	4.58	0.81	1.12	8.05	0.030	Significant
x_B_	7.48	0.81	4.01	10.95	0.011	Significant
x_C_	7.66	0.81	4.19	11.12	0.011	Significant
x_A_^2^	5.00	0.59	2.45	7.55	0.014	Significant
x_B_^2^	4.15	0.59	1.60	6.71	0.020	Significant
x_C_^2^	4.16	0.59	1.62	6.72	0.020	Significant
x_A_∙x_B_	−1.48	1.14	−6.39	3.42	0.322	Non-significant
x_A_∙x_C_	0.35	1.14	−4.55	5.25	0.788	Non-significant
x_B_∙x_C_	1.61	1.14	−3.28	6.52	0.292	Non-significant

**Table 5:** Effect estimates of the independent parameters on the efficiency of HSs isolation from lignite using birch ash extract (response Y_3_).

Source	Effect	Standard Error	Confidence Interval		*p*-Value	Remarks
			+95%	−95%		
x_A_	2.73	0.34	1.28	4.18	0.015	Significant
x_B_	2.93	0.34	1.47	4.38	0.013	Significant
x_C_	1.99	0.34	0.53	3.44	0.028	Significant
x_A_^2^	1.46	0.25	0.39	2.53	0.028	Significant
x_B_^2^	1.14	0.25	0.07	2.21	0.045	Significant
x_C_^2^	0.76	0.25	−0.30	1.84	0.091	Non-significant
x_A_∙x_B_	−0.32	0.48	−2.37	1.74	0.578	Non-significant
x_A_∙x_C_	0.41	0.48	−1.64	2.47	0.482	Non-significant
x_B_∙x_C_	0.48	0.48	−1.58	2.53	0.425	Non-significant

**Table 6:** Effect estimates of the independent parameters on the efficiency of HSs isolation from lignite using oak ash extract (response Y_4_).

Source	Effect	Standard Error	Confidence Interval		*p*-Value	Remarks
			+95%	−95%		
x_A_	3.15	0.52	0.91	5.39	0.026	Significant
x_B_	6.48	0.52	4.24	8.72	0.006	Significant
x_C_	3.56	0.52	1.32	5.80	0.021	Significant
x_A_^2^	3.23	0.38	1.58	4.88	0.014	Significant
x_B_^2^	4.35	0.38	2.70	6.00	0.008	Significant
x_C_^2^	2.10	0.38	0.45	3.75	0.032	Significant
x_A_∙x_B_	−2.98	0.74	−6.15	0.19	0.056	Non-significant
x_A_∙x_C_	−2.33	0.74	−5.50	0.84	0.087	Non-significant
x_B_∙x_C_	−0.24	0.74	−3.40	2.94	0.780	Non-significant

**Table 7:** Analysis of variance (ANOVA) for the polynomial models created.

Source	Sum of Squares (SS)	Degree of Freedom (df)	Mean Square (MS)	F-Value	*p*-Value
Response Y_1_					
Model	82.38	8	10.30	26.41	1.14 × 10^−4^
Residual	2.36	6	0.39		
Lack of fit	2.21	4	0.55	7.59	0.120
Pure error	0.15	2	0.07		
Response Y_2_					
Model	491.26	6	81.88	47.88	6.22 × 10^−6^
Residual	13.17	8	1.71		
Lack of fit	11.11	6	1.85	1.43	0.467
Pure error	2.60	2	1.30		
Response Y_3_					
Model	51.55	5	10.31	16.11	1.16 × 10^−4^
Residual	5.73	9	0.64		
Lack of fit	5.27	7	0.75	3.29	0.253
Pure error	0.46	2	0.23		
Response Y_4_					
Model	254.02	6	43.34	11.96	4.14 × 10^−4^
Residual	28.29	8	3.54		
Lack of fit	27.20	6	4.53	8.34	0.111
Pure error	1.09	2	0.54		

**Table 8:** The optimization results and corresponding experimental validation for the tested HSs isolation process variants.

Raw Material Type	Extractant Type	Optimal Conditions			Efficiency of HSs Extraction, %	
		x_A_, mW∙cm^−2^	x_B_, min	x_C_, °C	Predicted	Experimental
Peat	Birch ash extract	391	138	79	16.06	17.15
	Oak ash extract	320	126	70	37.52	38.71
Lignite	Birch ash extract	349	142	76	14.01	15.04
	Oak ash extract	309	116	67	27.34	29.13

**Table 9:** The results of the efficiency of HSs extraction from peat and lignite using NaOH solutions.

Raw Material Type	NaOH Concentration, mol∙dm^−3^	Efficiency of HSs Extraction, %
Peat	0.1	28.68
	0.5	57.15
Lignite	0.1	17.24
	0.5	31.02

**Table 10:** The results of CHNSO for the humic and fulvic samples.

Sample ^1^	Elemental Composition, at. %				
	C	H	N	S	O
HAs_Y_1_	32.37 ± 0.35	48.56 ± 0.55	2.06 ± 0.03	0.73 ± 0.01	16.28 ± 0.18
HAs_Y_2_	32.07 ± 0.35	47.90 ± 1.18	1.91 ± 0.03	0.92 ± 0.02	17.20 ± 0.70
HAs_Y_3_	35.94 ± 0.21	48.58 ± 0.27	0.61 ± 0.01	0.65 ± 0.02	14.22 ± 0.36
HAs_Y_4_	36.91 ± 1.48	45.44 ± 1.17	0.65 ± 0.08	0.81 ± 0.03	16.19 ± 0.80
FAs_Y_1_	29.83 ± 0.21	49.82 ± 0.34	3.03 ± 0.03	1.08 ± 0.08	16.24 ± 0.05
FAs_Y_2_	29.66 ± 1.39	47.86 ± 2.01	3.28 ± 0.12	1.06 ± 0.14	18.14 ± 0.52
FAs_Y_3_	32.56 ± 0.42	51.06 ± 1.16	0.92 ± 0.02	0.77 ± 0.13	14.69 ± 0.36
FAs_Y_4_	34.21 ± 1.14	47.97 ± 1.11	0.93 ± 0.01	0.85 ± 0.12	16.04 ± 0.66

^1^ Sample identifications: HAs_Y_1_—humic acids isolated from peat using birch ash extract; HAs_Y_2_—humic acids isolated from peat using oak ash extract; HAs_Y_3_—humic acids isolated from lignite using birch ash extract; HAs_Y_4_—humic acids isolated from lignite using oak ash extract; FAs_Y_1_—fulvic acids isolated from peat using birch ash extract; FAs_Y_2_—fulvic acids isolated from peat using oak ash extract; FAs_Y_3_—fulvic acids isolated from lignite using birch ash extract; FAs_Y_4_—fulvic acids isolated from lignite using oak ash extract.

**Table 11:** Relative percentage distributions of carbon atom types in HA and FA samples.

Sample	Percentage Distribution, %							
	Alkyl C (0–45 ppm)	O, N-Alkyl C (45–60 ppm)	O-Alkyl C (60–91 ppm)	Dioxyalkyl C (91–110 ppm)	Aromatic C (110–142 ppm)	O,N-Aromatic C (142–156 ppm)	Carboxyl-C (156–186 ppm)	Carbonyl-C (186–230 ppm)
HAs_Y_1_	23.01	7.01	9.04	4.68	22.06	8.44	16.51	9.25
HAs_Y_2_	24.73	4.58	6.36	4.29	24.87	7.63	15.73	11.81
HAs_Y_3_	31.30	2.98	4.19	4.46	32.44	3.47	10.35	10.81
HAs_Y_4_	26.16	3.33	5.67	4.25	32.71	6.92	9.68	11.28
FAs_Y_1_	24.76	5.71	10.71	6.72	22.52	2.73	17.91	8.94
FAs_Y_2_	25.40	5.55	10.15	6.69	21.26	6.33	16.81	7.81
FAs_Y_3_	27.07	3.69	9.39	4.23	24.57	3.78	19.86	7.41
FAs_Y_4_	27.16	4.02	8.36	4.98	23.11	5.53	17.81	9.03

## Data Availability

The data presented in this study are available on request from the corresponding author.
